# Supplementary material for: Murine hematopoietic progenitor cell lines with erythroid and megakaryocyte potential
Source: Nat Commun. 2025 Aug 7;16:7283. doi: 10.1038/s41467-025-62668-z (PMC12331996; doi:10.1038/s41467-025-62668-z)
Supplement: Supplementary file 1 — Supplementary Information [file 41467_2025_62668_MOESM1_ESM.pdf]

a

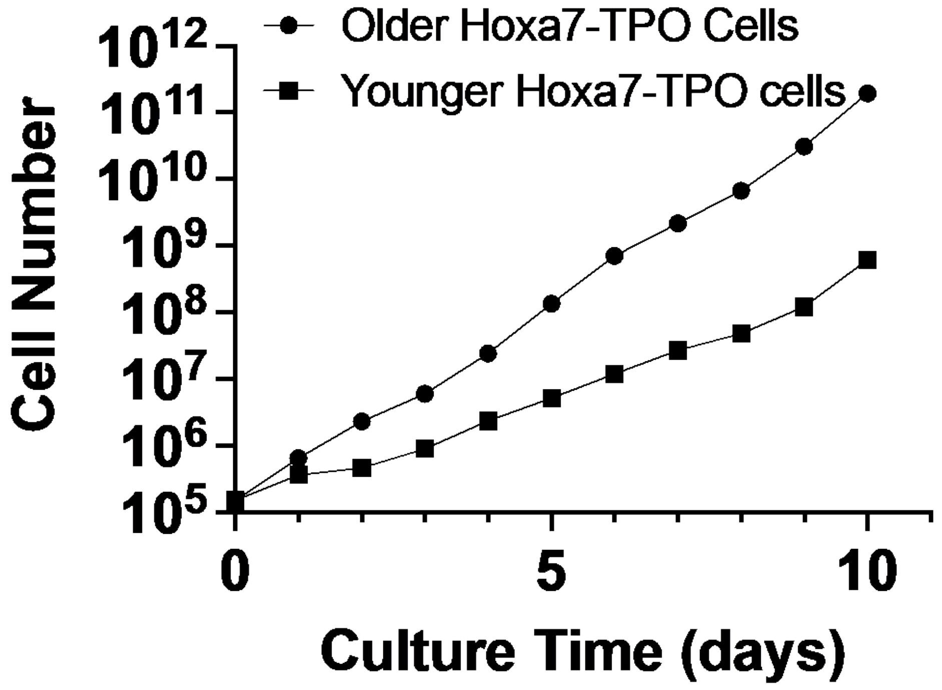

b

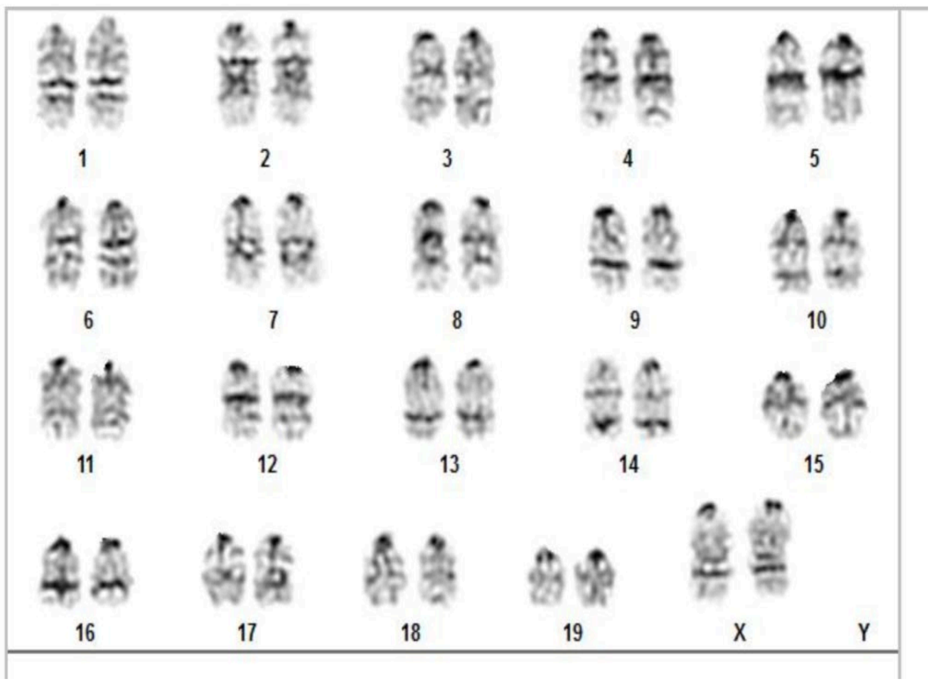

Supplementary Fig. 1 ***In vitro*** **proliferation and karyotype analysis of Hoxa7-TPO cells that were cultured for extended periods of time.**

a Comparison of growth characteristics of Hoxa7-TPO cells that were cultured for 4-6 weeks (‘younger’ Hoxa7-TPO cells) and Hoxa7-TPO cells that were continuously cultured for one year (‘older’ Hoxa7-TPO cells). Source data are provided as a Source Data file.

b Karyotype analysis of Hoxa7-TPO cells after one year of continuous cell culture.

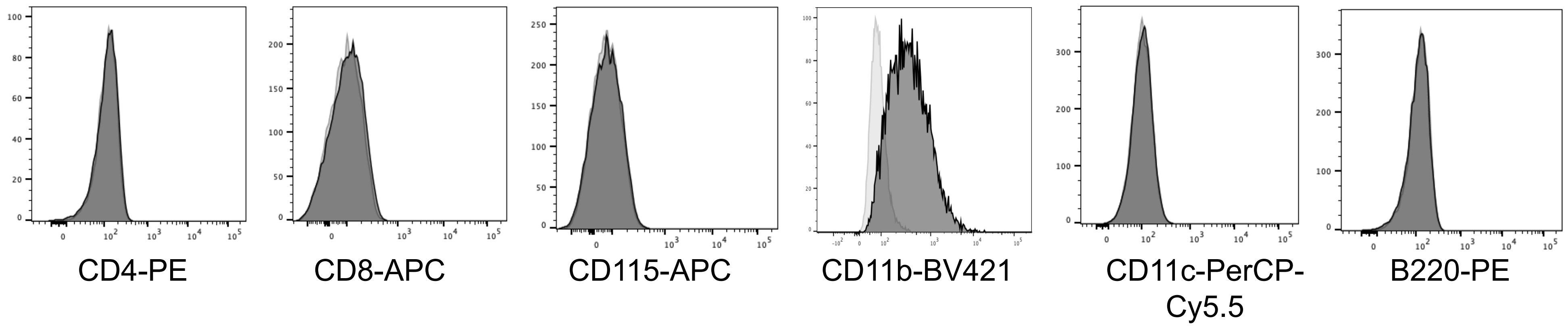

Supplementary Fig. 2 Flow cytometry analysis of Hoxa7-TPO cells.

Non-differentiated Hoxa7-TPO cells were stained with indicated antibodies and analyzed by flow cytometry. Dark grey, specific antibodies; light grey, isotype controls.

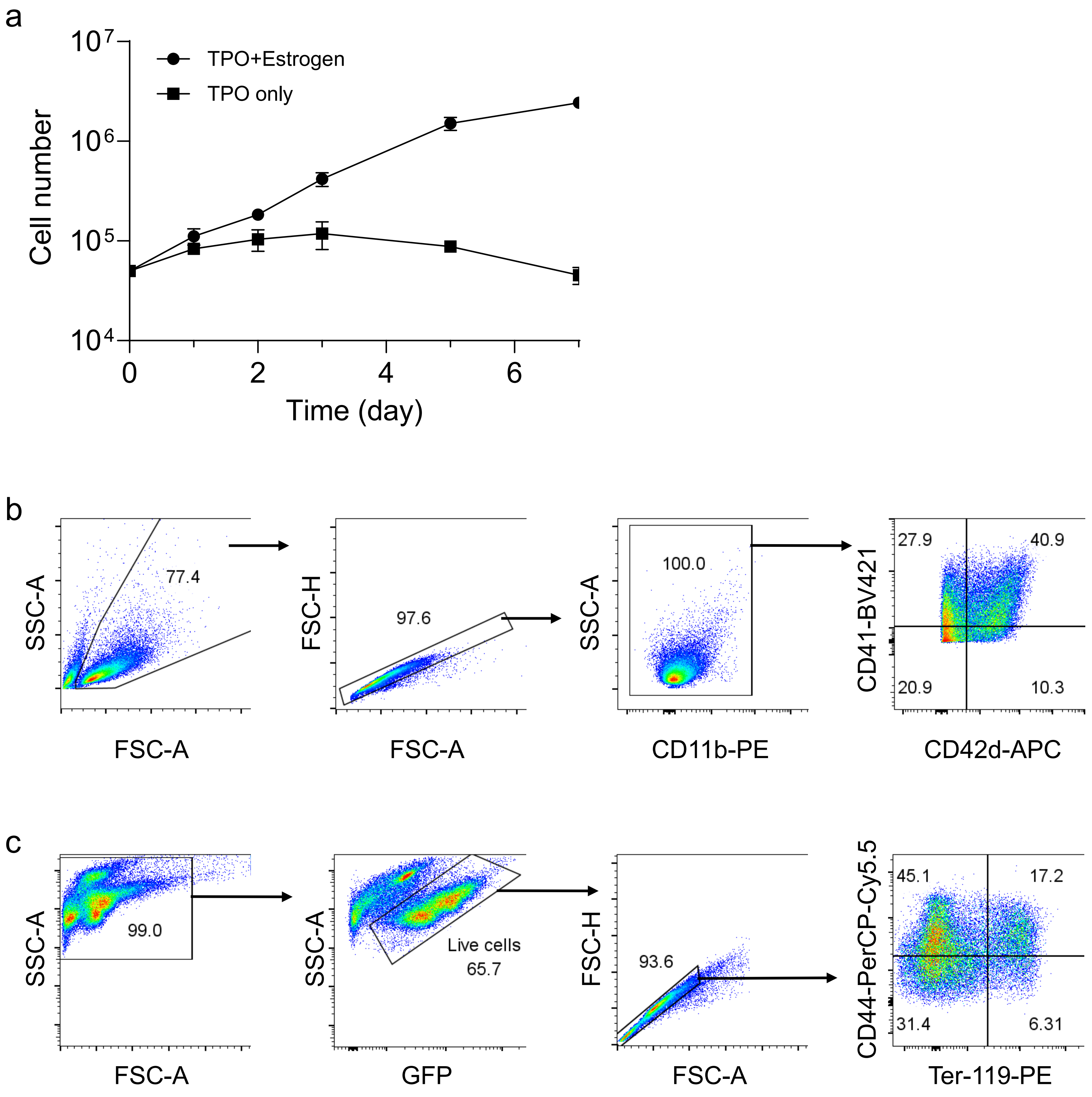

Supplementary Fig. 3 Estrogen-dependent growth characteristics of Hoxa7-TPO cells and flow cytometry-based gating strategy for analysis of *in vitro* differentiated Hoxa7-TPO cells.

a Cell numbers of Hoxa7-TPO cells cultured with TPO (20 ng/ml) in the presence or absence of exogenous estrogen. Data represent mean ± SD from n = 5 technical replicates at each indicated time point. Source data are provided as a Source Data file.

b Gating strategy for analysis of *in vitro* MK cell differentiation of Hoxa7-TPO cells.

c Gating strategy for analysis of *in vitro erythroid* differentiation of Hoxa7-TPO cells.

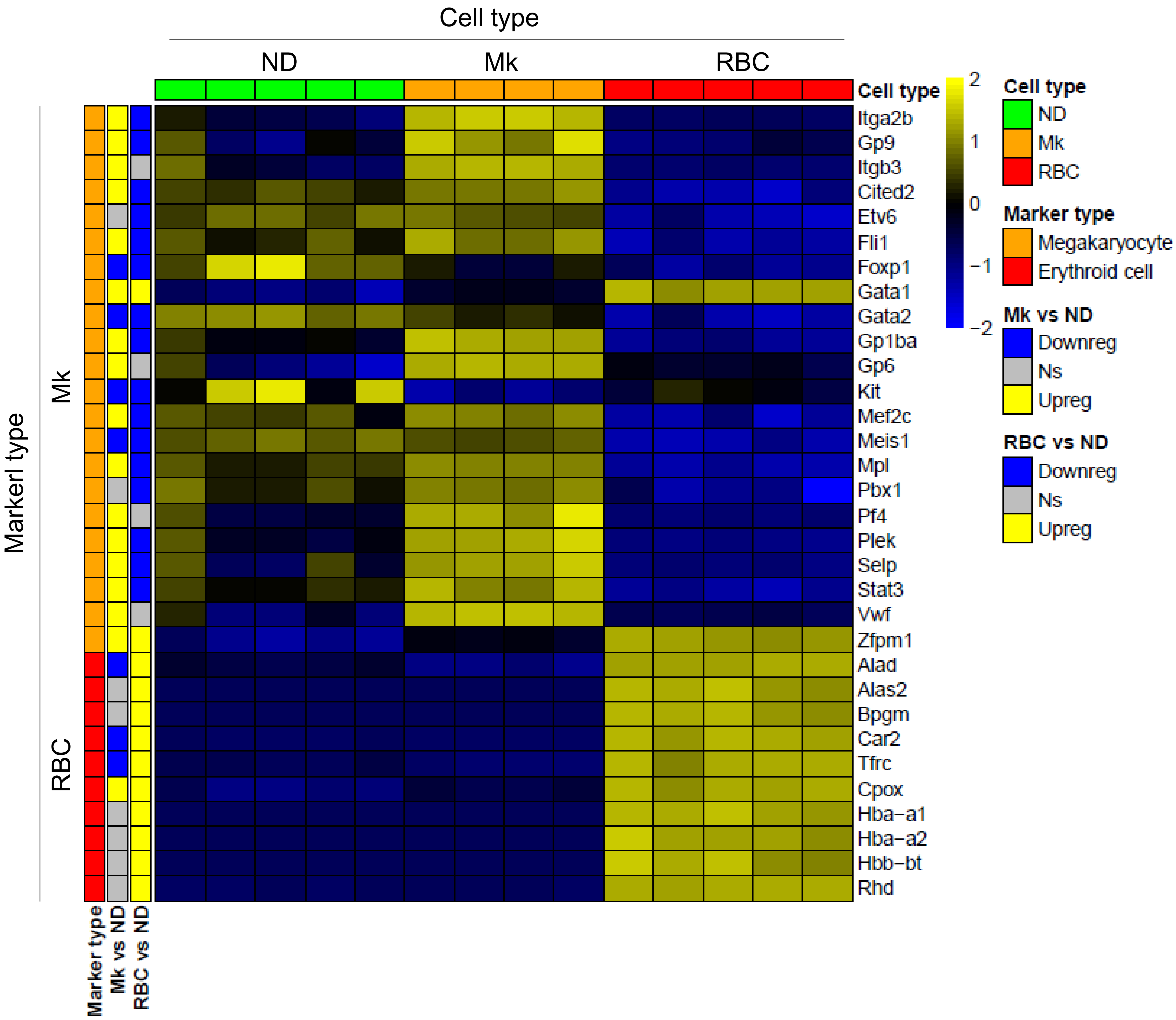

Supplementary Fig. 4 RNAseq-based heat map of marker genes encoding lineage-defining factors.

Analysis is based on RNAseq from non-differentiated Hoxa7-TPO cells, Hoxa7-TPO cells that were differentiated for 4 days *in vitro* in the presence of TPO and Hoxa7-TPO-derived reticulocytes that were isolated from adoptively transferred mice. Experimentally defined marker genes were drawn from the updated, manually curated (scRNA-seq-based) CellMarker 2.0 database (PMID: 36300619).

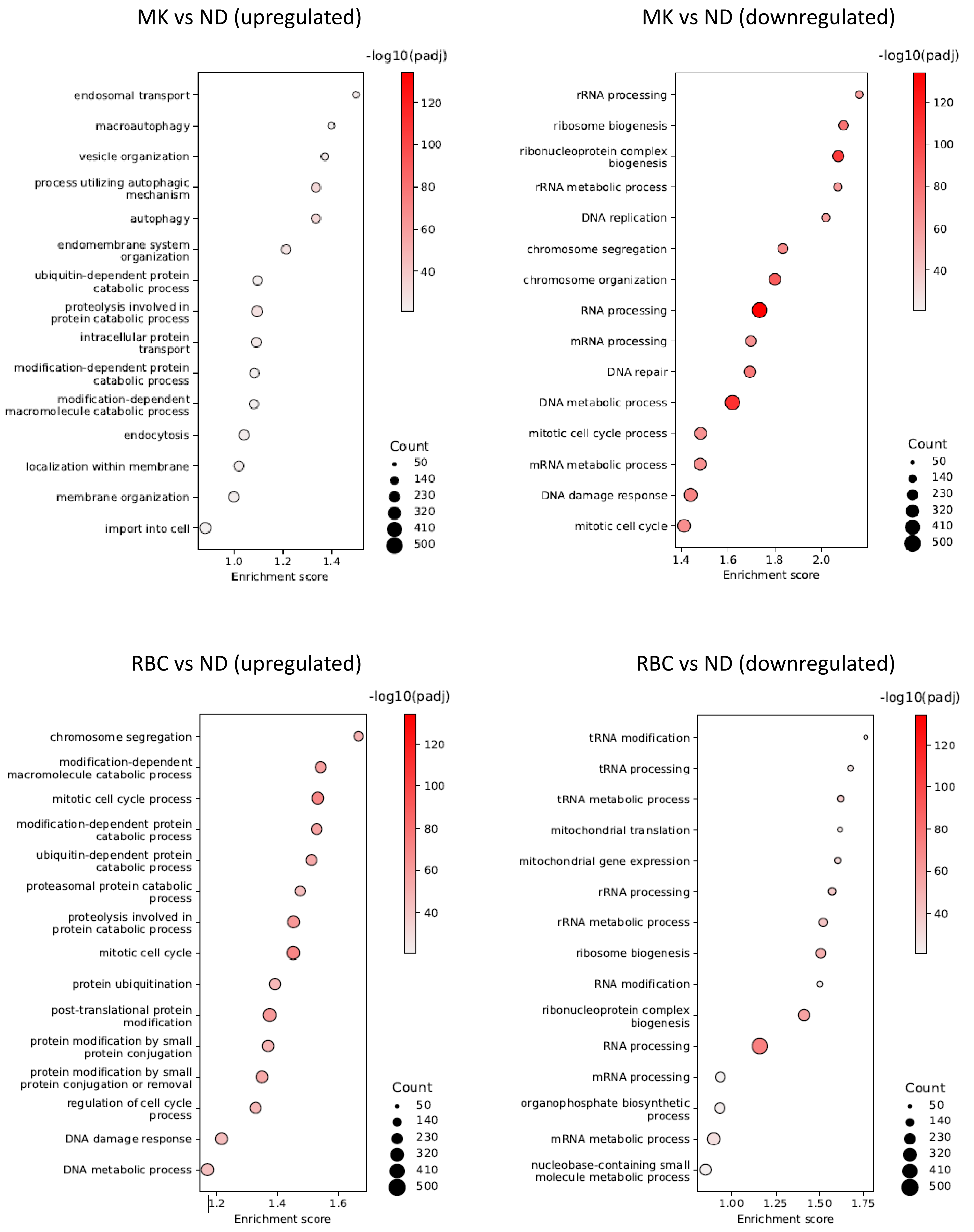

Supplementary Fig. 5 RNAseq-based gene ontology analysis based on up- and down-regulated genes in MK and reticulocytes vs. non-differentiated cells (ND).

For each condition (non-differentiated Hoxa7-TPO cells, Hoxa7-TPO cells that were differentiated for 4 days *in vitro* in the presence of TPO and Hoxa7-TPO-derived reticulocytes that were isolated from adoptively transferred mice), the top 15 most significantly enriched gene ontology “Biological Process” terms are shown (Fisher’s exact test with Benjamini-Hochberg correction), ranked by decreasing enrichment score.

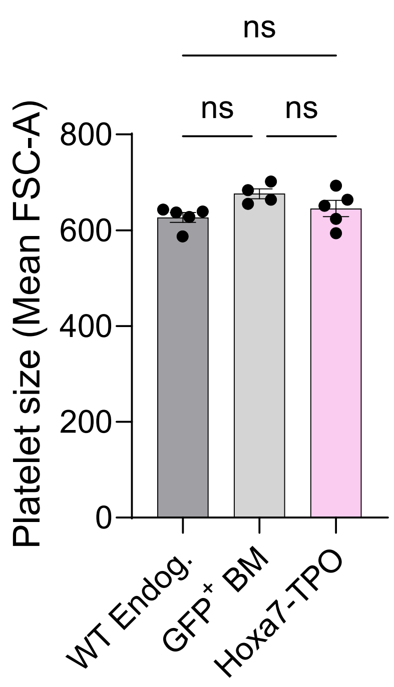

Supplementary Fig. 6 Cell size of Hoxa7-TPO- and BM-derived platelets.

Flow cytometry-based forward scatter analysis of platelets in lethally irradiated mice that were adoptively transferred with BM WT helper cells and Hoxa7-TPO cells (WT endog., Hoxa7-TPO) or GFP$^+$ BM cells (GFP$^+$ BM) and analyzed 7 days later. Data represent mean ± S.E. from n = 5 independent experiments for WT Endog. BM and Hoxa7-TPO, and n = 4 for GFP$^+$ BM. Ns, not significant (P > 0.05), one-way ANOVA. Source data are provided as a Source Data file.

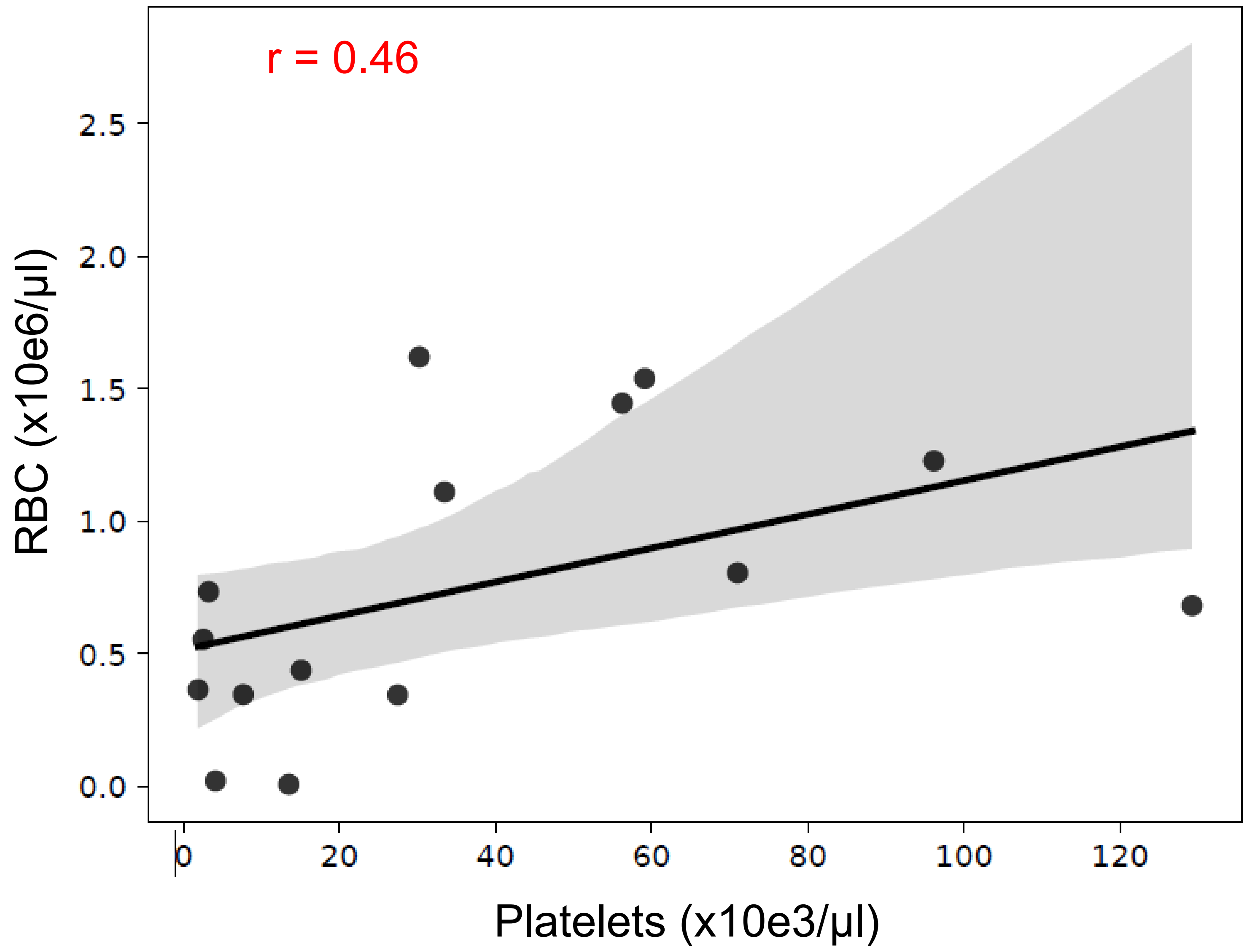

Supplementary Fig. 7 Correlation analysis of RBC and platelets produced from Hoxa7-TPO clones *in vivo.* Correlation of RBC and platelet counts from the PB of mice that were adoptively transferred with 15 clones derived from Hoxa7-TPO cells. Each symbol represents data obtained from individual mice transferred with one individual clone. Regression line, 95% confidence interval (shaded) and Pearson correlation index (r, in red) are indicated. Source data are provided as a Source Data file.

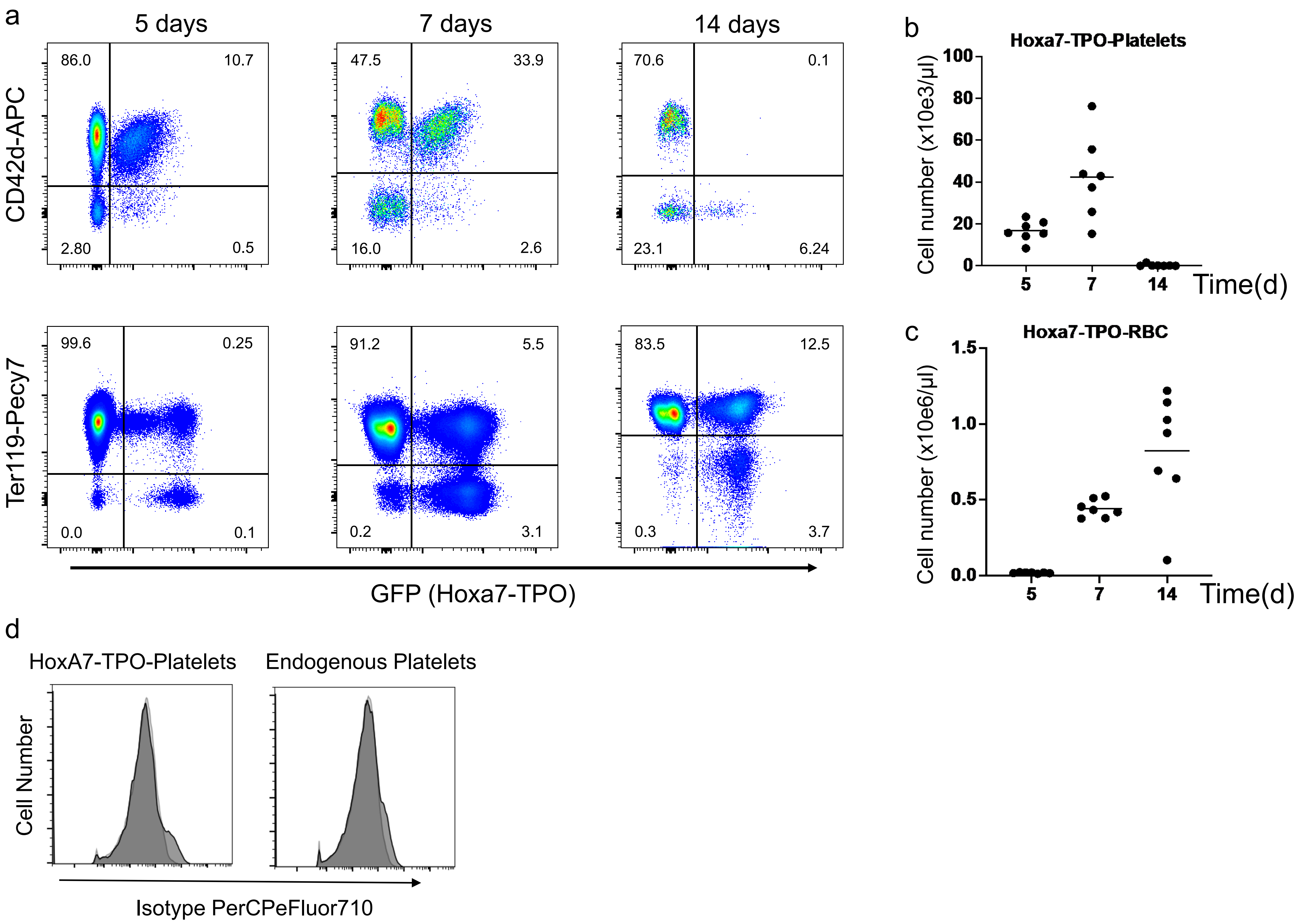

Supplementary Fig. 8 ***In vivo*** **potential of Hoxa7-TPO cells after long-term storage in liquid nitrogen and CD62L isotype controls for thrombin-activated platelets.**

a Flow cytometry analysis of the PB of mice that were adoptively transferred with GFP-expressing Hoxa7-TPO cells (at indicated time points) that had been stored in liquid nitrogen for four years.

b,c Quantitative analysis of Hoxa7-TPO-derived platelets (b) and RBC (c) described in (a). Symbols represent individually transferred mice (n = 7). Source data are provided as a Source Data file.

d Flow cytometry of thrombin-treated platelets using isotype control antibodies. Platelet-rich plasma was prepared from mice six days after adoptive transfer with GFP+ Hoxa7-TPO cells and BM-derived helper cells, followed by thrombin treatment (2U/ml) for 20 min. Light grey, non-treated cells; dark gray, thrombin-treated cells.

Mice group	% platelets (mean ± SEM)	AUC (mean ± SEM)
Non-treated		
CD41+	5.98 ± 1.09	7,703 ± 200
BM only		
CD41+	3.89 ± 0.40	4,417 ± 184
BM+Hoxa7-TPO		
CD41+	1.60 ± 0.52 (33.7 %)	18,063 ± 917 (44.3%)
CD41+ GFP+	3.15 ± 1.50 (66.3 %)	22,748 ± 929 (55.7 %)

Supplementary Fig. 9 Relative size of cremaster arteriole thrombi and platelet number post laser injury in *Mpl*$^{-/-}$ mice.

Flow cytometry studies determined the percent of cells present in the peripheral blood that are either CD41^{+} or CD41^{+}GFP^{+} platelets in the mice at the time of the thrombosis study. The size of the thrombi post-laser injury in cremaster arterioles are shown in relative area-under-the-curve (AUC). Both results are mean ±1 SEM. BM only = *Mpl*$^{-/-}$ mice receiving WT BM marrow transplant only. BM+Hoxa7-TPO = *Mpl*$^{-/-}$ mice transplanted with both WT marrow and non-differentiated Hoxa7-TPO cells. The percentage of endogenous (CD41^{+}) and Hoxa7-TPO-derived platelets (CD41^{+} GFP^{+}) contributing to thrombi are depicted in red in brackets. All arms had 4 mice studied.

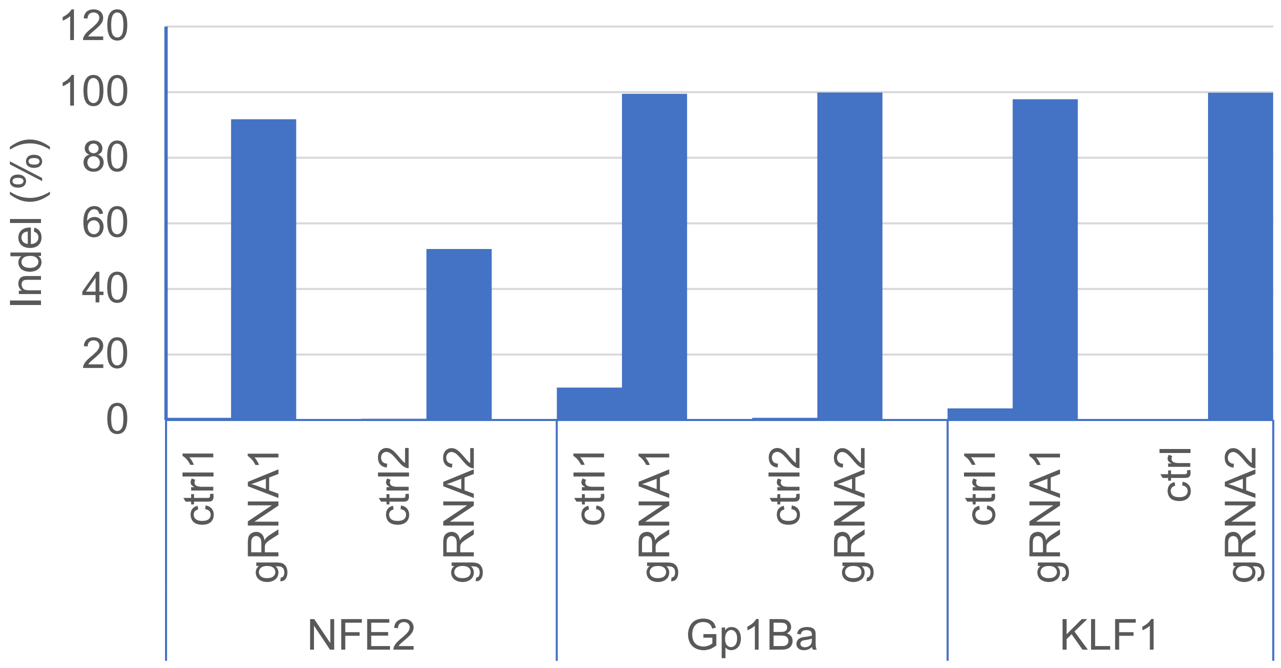

Supplementary Fig. 10 gRNA targeting efficiency

On-target DNA editing efficiency (indel frequency) of sgRNA-transduced Hoxa7-TPO cells was determined by next-generation sequencing. Source data are provided as a Source Data file.

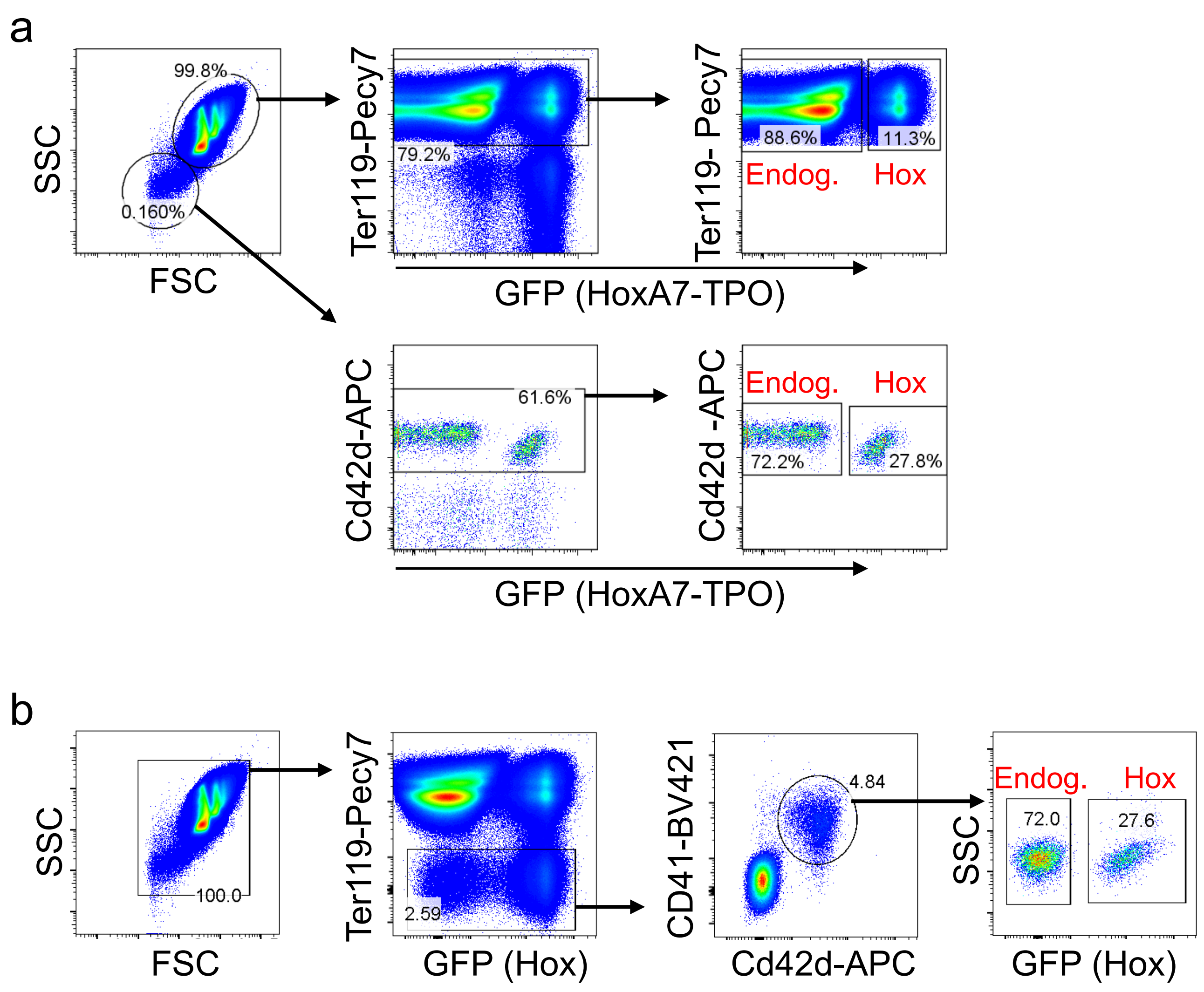

Supplementary Fig. 11 Gating strategy for platelets

a Flow cytometry-based gating strategy for standard assays based on adoptive transfer of Hoxa7-TPO cells.

b Flow cytometry-based gating strategy for analysis of large platelets as obtained upon genetic deletion of GP1BA.

gene-gRNA Name	gRNA target sequence	target-seq-F-primer	target-seq-R-primer
NFE2_g1	TGGTATGGCTACATTACCAT	CACTCTTTCCCTACACGACGCTCTTCCGATCTGCCACTGCCACCTCCAACAT	GTGACTGGAGTTCAGACGTGTGCTCTTCCGATCCACTGGCAGCCCAATGTCCA
NFE2_g2	AAGTTAACTATCTTGTCCGT	CACTCTTTCCCTACACGACGCTCTTCCGATCTTCCGGTCCAGTTCGGGCTAA	GTGACTGGAGTTCAGACGTGTGCTCTTCCGATCCCACCTTGTTCTTGCCCCGT
Gp1Ba_g1	GACAGGCGCATTGTCCAAGT	CACTCTTTCCCTACACGACGCTCTTCCGATCTCCACGCCAACAGCTGGTACT	GTGACTGGAGTTCAGACGTGTGCTCTTCCGATCCTTGTGGCAGGGACGTCAGG
Gp1Ba1_g2	TGGCTTCGCACAATACCAAA	CACTCTTTCCCTACACGACGCTCTTCCGATCTATTGCGTGAGCTGCCCTCTG	GTGACTGGAGTTCAGACGTGTGCTCTTCCGATCTTCCTGGAGCCAGTGACGGA
KLF1_g1	GCTCGCGAGTCGGAGTACGA	GGCTCCGACACTCTTTCCCTACACGACGCTCTTCCGATCTGGCTCCGAGGAGCACACAAGGGAGCACACAAG	GTGACTGGAGTTCAGACGTGTGCTCTTCCGATCCCCGCGAAAGAGCTGGAAGT
KLF1_g2	GAGGGACGTGACCTGTGCGT	CACTCTTTCCCTACACGACGCTCTTCCGATCTCAAGCAGTACGCTCCCTCCC	GTGACTGGAGTTCAGACGTGTGCTCTTCCGATCAGAGACTCAGGCGGCTCGAA

Supplementary Table 1

Sequence information of sgRNA targets and primers used for analysis of targeting efficiency
