# Supplementary material for: Murine hematopoietic progenitor cell lines with erythroid and megakaryocyte potential
Source: Nat Commun. 2025 Aug 7;16:7283. doi: 10.1038/s41467-025-62668-z (PMC12331996; doi:10.1038/s41467-025-62668-z)
Supplement: Supplementary file 2 — Description of Additional Supplementary Files [file 41467_2025_62668_MOESM2_ESM.pdf]

Description of Additional supplementary files

Supplementary Data 1.

List of differentially expressed genes in Hoxa7- TPO cells and Hoxa7-TPO-derived MK and reticulocytes. Gene expression of non-differentiated Hoxa7-TPO cells and Hoxa7-TPO cells that were differentiated for four days in vitro (MK) or were isolated ex vivo 7 days after adoptive transfer (reticulocytes) was analyzed by RNAseq. Differentially expressed genes (adj. Pval ≤ 0.05, LogFC ≥ 5) are shown. Differential expression genes were tested with limma-voom in R, which is a moderated two sided t test. Results were adjusted for multiple comparisons by the Benjamini-Hochberg method.

Supplementary Data 2.

Differentially expressed genes in Hoxa7-TPO-derived MK and reticulocytes vs. nondifferentiated Hoxa7-TPO cells as used for analysis of lineage-defining factors and gene ontology analysis. Differential expression analysis was performed using DESeq2 with the two-sided Wald test. Results were adjusted for multiple comparisons using the BenjaminiHochberg method.

Supplementary Data 3.

Reagents and antibodies used in this study.
