# Supplementary material for: Murine hematopoietic progenitor cell lines with erythroid and megakaryocyte potential
Source: Nat Commun. 2025 Aug 7;16:7283. doi: 10.1038/s41467-025-62668-z (PMC12331996; doi:10.1038/s41467-025-62668-z)
Supplement: Supplementary file 6 — Reporting Summary [file 41467_2025_62668_MOESM6_ESM.pdf]

nature portfolio

Corresponding author(s): Hans Haecker

Last updated by author(s): Jul 3, 2025

Reporting Summary

Nature Portfolio wishes to improve the reproducibility of the work that we publish. This form provides structure for consistency and transparency in reporting. For further information on Nature Portfolio policies, see our Editorial Policies and the Editorial Policy Checklist.

Statistics

For all statistical analyses, confirm that the following items are present in the figure legend, table legend, main text, or Methods section.

n/a	Confirmed	
☐	☒	The exact sample size (*n*) for each experimental group/condition, given as a discrete number and unit of measurement
☐	☒	A statement on whether measurements were taken from distinct samples or whether the same sample was measured repeatedly
☐	☒	The statistical test(s) used AND whether they are one- or two-sided<br>*Only common tests should be described solely by name; describe more complex techniques in the Methods section.*
☒	☐	A description of all covariates tested
☐	☒	A description of any assumptions or corrections, such as tests of normality and adjustment for multiple comparisons
☐	☒	A full description of the statistical parameters including central tendency (e.g. means) or other basic estimates (e.g. regression coefficient) AND variation (e.g. standard deviation) or associated estimates of uncertainty (e.g. confidence intervals)
☐	☒	For null hypothesis testing, the test statistic (e.g. *F*, *t*, *r*) with confidence intervals, effect sizes, degrees of freedom and *P* value noted<br>*Give P values as exact values whenever suitable.*
☒	☐	For Bayesian analysis, information on the choice of priors and Markov chain Monte Carlo settings
☒	☐	For hierarchical and complex designs, identification of the appropriate level for tests and full reporting of outcomes
☐	☒	Estimates of effect sizes (e.g. Cohen's *d*, Pearson's *r*), indicating how they were calculated

Our web collection on statistics for biologists contains articles on many of the points above.

Software and code

Policy information about availability of computer code

Data collection	For flow cytometry experiments data was collected using Becton Dickinson (BD) FACSDiva software (v8.0.1 or v9.0) or SpectroFlo (v3.3.0).
Data analysis	Data analysis was performed using the following software:<br>FlowJo (v9 and v10), GraphPad Prism (v10.4.1), ImageJ (v1.54p), Partek Genomics Suite 6.6 , TrimGalore (v0.6.10), STAR aligner (v2.7.11a), R package DESeq2 (v1.42.1).<br>The code used for the over-representation analysis is available at Github: https://github.com/mmarchetti90/project_setup_assistant/blob/main/code_base/term_enrichment/ora/enrichment_analysis_1.py.

For manuscripts utilizing custom algorithms or software that are central to the research but not yet described in published literature, software must be made available to editors and reviewers. We strongly encourage code deposition in a community repository (e.g. GitHub). See the Nature Portfolio guidelines for submitting code & software for further information.

Data

Policy information about availability of data

All manuscripts must include a data availability statement. This statement should provide the following information, where applicable:

- Accession codes, unique identifiers, or web links for publicly available datasets
- A description of any restrictions on data availability
- For clinical datasets or third party data, please ensure that the statement adheres to our policy

Relevant data supporting the findings of this study are available upon request from the corresponding author, respectively have been deposited at NCBI GEO (GSE244976).

Research involving human participants, their data, or biological material

Policy information about studies with human participants or human data. See also policy information about sex, gender (identity/presentation), and sexual orientation and race, ethnicity and racism.

Reporting on sex and gender	*Use the terms sex (biological attribute) and gender (shaped by social and cultural circumstances) carefully in order to avoid confusing both terms. Indicate if findings apply to only one sex or gender; describe whether sex and gender were considered in study design; whether sex and/or gender was determined based on self-reporting or assigned and methods used. Provide in the source data disaggregated sex and gender data, where this information has been collected, and if consent has been obtained for sharing of individual-level data; provide overall numbers in this Reporting Summary. Please state if this information has not been collected. Report sex- and gender-based analyses where performed, justify reasons for lack of sex- and gender-based analysis.*
Reporting on race, ethnicity, or other socially relevant groupings	*Please specify the socially constructed or socially relevant categorization variable(s) used in your manuscript and explain why they were used. Please note that such variables should not be used as proxies for other socially constructed/relevant variables (for example, race or ethnicity should not be used as a proxy for socioeconomic status). Provide clear definitions of the relevant terms used, how they were provided (by the participants/respondents, the researchers, or third parties), and the method(s) used to classify people into the different categories (e.g. self-report, census or administrative data, social media data, etc.) Please provide details about how you controlled for confounding variables in your analyses.*
Population characteristics	*Describe the covariate-relevant population characteristics of the human research participants (e.g. age, genotypic information, past and current diagnosis and treatment categories). If you filled out the behavioural & social sciences study design questions and have nothing to add here, write "See above."*
Recruitment	*Describe how participants were recruited. Outline any potential self-selection bias or other biases that may be present and how these are likely to impact results.*
Ethics oversight	*Identify the organization(s) that approved the study protocol.*

Note that full information on the approval of the study protocol must also be provided in the manuscript.

Field-specific reporting

Please select the one below that is the best fit for your research. If you are not sure, read the appropriate sections before making your selection.

☒ Life sciences ☐ Behavioural & social sciences ☐ Ecological, evolutionary & environmental sciences

For a reference copy of the document with all sections, see nature.com/documents/nr-reporting-summary-flat.pdf

Life sciences study design

All studies must disclose on these points even when the disclosure is negative.

Sample size	Sample sizes were not predetermined by formal statistical methods; instead, they were chosen according to field standards or prior laboratory experience. For some experiments, effect size metrics such as Cohen's d (for t-tests) were calculated post hoc to aid in interpreting the magnitude of observed effects.
Data exclusions	No data were excluded.
Replication	All replication experiments were successful.
Randomization	For in vivo experiments, mice were randomly allocated to experimental groups. Randomization was not relevant to in vitro experiments.
Blinding	Investigators were not blinded to group allocations, as blinding was not applicable to the experimental design.

Reporting for specific materials, systems and methods

We require information from authors about some types of materials, experimental systems and methods used in many studies. Here, indicate whether each material, system or method listed is relevant to your study. If you are not sure if a list item applies to your research, read the appropriate section before selecting a response.

Materials & experimental systems

n/a	Involved in the study
☐	☒ Antibodies
☐	☒ Eukaryotic cell lines
☒	☐ Palaeontology and archaeology
☐	☒ Animals and other organisms
☒	☐ Clinical data
☒	☐ Dual use research of concern
☒	☐ Plants

Methods

n/a	Involved in the study
☒	☐ ChIP-seq
☐	☒ Flow cytometry
☒	☐ MRI-based neuroimaging

Antibodies

Antibodies used

From Thermofisher Scientific:

TER-119-Pecy7 (#25-5921-82)
Ter-119-PE (#12-5921-82)
CD16/CD32-PE (93; #12-0161-82)
CD11c-PerCP-cy5.5 (N418; 45-0114-82)
CD42d-APC (1C2; #17-0421-82)
B220-PE (RA3-6B2; #12-0452-82)
CD62P-PerCP-eFluor710 (Psel.KO2.3; # 46-0626-82)
CD115-APC (AFS98; 17-1152-82)
CD11b-BV421 (M1/70; #404-0112-82)
donkey anti-mouse IgG- Alexa Fluor™ 488 (#A-21202)
donkey anti-rabbit IgG- Alexa Fluor™ 488 (#A-21206)

From BD:

CD8a-APC (53-6.7; #553035)
CD4-APC (RM4-5; #561091)
CD117-APC (2B8; # 561074)
CD71-PE (C2; #561937)

From Biolegend:

Sca-1-PE (E13-161.7; #122507)
LY-6G-Pecy7 (1A8; #127617)
Ly6C-APC (HK1.4; #128015)
CD150-APC (TC15-12F12.2; #115909)
CD105-Pecy7 (MJ7/18; #120409)
CD44-PerCP-Cy5.5 (IM7; #103031)
CD42d- Percp-Cy5.5 (1C2; #148508)
CD41 BV421 (MWReg30; #133912)
CD34-PE (SA376A4; #152203)

AAnti-β1-tubulin (Sigma; #T8328)
Anti-VWF (Santa Cruz Biotech; # sc-365712)

Validation

Validation information for the antibodies listed above is available on the manufacturers' websites using the corresponding catalogue numbers.

Eukaryotic cell lines

Policy information about cell lines and Sex and Gender in Research

Cell line source(s)

HEK293T cells for virus production were obtained from Takara (632180). Hoxa7-TPO cells were generated from female mice.

Authentication	HEK293T cells were not further authenticated beyond Takara's certification. The detailed characterization of Hoxa7-TPO cells is key part of the manuscript.
Mycoplasma contamination	The cell lines were regularly tested for mycoplasma and found negative.
Commonly misidentified lines (See ICLAC register)	No commonly misidentified lines were used.

Animals and other research organisms

Policy information about studies involving animals; ARRIVE guidelines recommended for reporting animal research, and Sex and Gender in Research

Laboratory animals	C57BL/6 mice were purchased from Jackson Laboratories and were either used directly in experiments or bred in the animal facility at the University of Utah. PF4-Cre and ROSA26iDTR mice were obtained from Jackson Laboratories and crossed in the University of Utah's animal facility. H2K-GFP mice were kindly provided by Dr. Derek Persons and were housed and bred at the University of Utah's animal facility. Mpl-/- mice were sourced from Dr. Wei Tong at the Children's Hospital of Philadelphia. All mice were closely monitored by resident veterinarians to ensure their well-being. The dark/light cycle, temperature, and humidity were centrally controlled.
Wild animals	N/A
Reporting on sex	Female mice and cell lines derived from female mice were used for this study.
Field-collected samples	N/A
Ethics oversight	All mouse studies were carried out in accordance with protocols approved by the Institutional Animal Care and Use Committees at the St. Jude Children's Research Hospital, the University of Utah and the Children's Hospital of Philadelphia.

Note that full information on the approval of the study protocol must also be provided in the manuscript.

Plants

Seed stocks	N/A
Novel plant genotypes	N/A
Authentication	N/A

Flow Cytometry

Plots

Confirm that:

- [x] The axis labels state the marker and fluorochrome used (e.g. CD4-FITC).
- [x] The axis scales are clearly visible. Include numbers along axes only for bottom left plot of group (a 'group' is an analysis of identical markers).
- [x] All plots are contour plots with outliers or pseudocolor plots.
- [x] A numerical value for number of cells or percentage (with statistics) is provided.

Methodology

Sample preparation	Described in Methods section.
Instrument	BD LSRFortessa; BD LSRFortessa X-20; Cytek Aurora
Software	Data collection: BD FACSDiva V. 9.0; BD FACSDiva V. 8.0.1; SpecFlo V. 3.3.0<br>Data analysis: FlowJoe (various version)
Cell population abundance	Cell populations abundances are provided where relevant by quantification.

Gating strategy

All gating strategies are detailed in the supplementary section of the paper, including frequencies of gated populations.

☒ Tick this box to confirm that a figure exemplifying the gating strategy is provided in the Supplementary Information.
